# African Vaccinology Network (AfVANET): an African network by African scientists

**DOI:** 10.11604/pamj.2020.37.66.21688

**Published:** 2020-09-16

**Authors:** David Dazhia Lazarus, Funmilayo Ibitayo Deborah Afolayan, Gezahegne Mamo, Jerome Nyhalah Dinga, Jones Akinbobola, Kwabena Obeng Duedu, Nefefe Tshifhiwa, Tesfaye Kassa, Vish Nene, Yakhya Dieye, Mustapha Oumouna

**Affiliations:** 1 Faculty of Veterinary Science, University of Pretoria, Pretoria, South Africa,; 2 National Veterinary Research Institute, Vom, Plateau, Nigeria,; 3 Department of Zoology, University of Ibadan, Ibadan, Nigeria,; 4 College of Veterinary Medicine and Agriculture, Addis Ababa University, Addis Ababa, Ethiopia,; 5 Biotechnology Unit, University of Buea, Buea, Cameroon,; 6 Faculty of Veterinary Medicine, Department of Veterinary Medicine, University of Abuja, Abuja, Nigeria,; 7 Department of Biomedical Sciences, University of Health and Allied Sciences, Ho, Ghana,; 8 ARC-Onderstepoort Veterinary Research, Pretoria, South Africa,; 9 School of Medical Laboratory Science, Institute of Health, Jimma University, Jimma, Ethiopia,; 10 International Livestock Research Institute, Nairobi, Kenya,; 11 University Cheikh Anta Diop and Pasteur Institute, Dakar, Senegal,; 12 Faculty of Sciences, University of Médéa, Médéa, Algeria

**Keywords:** African network, African scientists, vaccine research, diseases

## To the editors of the Pan African Medical Journal

We write to introduce the African Vaccinology Network (AfVANET) as a new network of African research scientists involved in vaccine research and development (R&D) for human and animal diseases. The goal of this network is to promote and build capacity for early phase vaccine R&D in Africa in order to stimulate the development of innovative solutions to combat diseases that affect the continent. This will be achieved by bringing together different stakeholders in vaccinology and related sciences in Africa to identify and prioritise gaps in vaccine development for both human and animal diseases and to promote sound ethics, biosafety, biosecurity and animal welfare practices, facilitate the mobility of students and early career researcher between research institutions and universities in Africa through south-south collaborations and address the gender imbalance in the scientific workforce. The African continent has for decades suffered the social and economic consequences of several infectious diseases. Examples include the recent spate of infectious disease outbreaks as seen with the Ebola outbreak in the Democratic Republic of Congo (DRC) [1], Lassa fever in Nigeria and Liberia [2,3], cholera in some parts of Nigeria [4], yellow fever in Angola, the DRC, South Sudan and Nigeria [5-7] and measles and Rift Valley fever in some African countries [8,9]. In addition, there are continual loses in livestock productivity due to, e.g. African trypanosomiasis, ticks and tick-borne diseases and lack of access to global markets due to the presence of trans-boundary diseases such as contagious bovine pleuropneumonia, foot-and-mouth disease and African swine fever. Hence, there is an urgent need for a sound framework for R&D towards developing novel and effective vaccines for human and animal diseases.

In spite of many public health threats, drug and vaccine development against diseases endemic to Africa has on most occasions been initiated and pioneered by expatriate researchers. Often, the need to develop these products is only seen when the diseases cause a significant threat to people and economies for which industries foresee a potential to make profit. This has led to a huge gap in the availability of affordable and effective drugs and vaccines for many diseases of the African continent. The concept of AfVANET was born out of the need to address African problems by African researchers during the keystone symposium “New Approaches to Vaccines for Human and Veterinary Tropical Diseases” held in Cape Town, South Africa 22-26^th^ May 2016. This network was mainly motivated by the observation that while African countries suffer the most from infectious diseases discussed during the conference, the research and innovation used to tackle these diseases mostly come from outside the continent. We therefore discussed and stressed the need for a better involvement of African scientists in finding solutions to infectious diseases that affect the health and wellbeing of people on the continent. Indeed, the knowledge of the field by local scientists constitutes an invaluable asset that can be mobilized in the fight against human and animal infectious diseases. The AfVANET had its first maiden scientific workshop supported by the International Veterinary Vaccinology Network (IVVN) with the theme: a roadmap for promoting effective vaccine research and development in Africa from 19-20^th^ March, 2019 at the International Livestock Research Institute, (ILRI) campus, Nairobi, Kenya [10].

More information on AfVANET is available via our website. AfVANET is managed by a steering committee that represents different regions of Africa: Prof. Mustapha Oumouna, general coordinator and North Africa coordinator; Dr. Jerome Nyhalah Dinga, Central Africa coordinator; Dr. Nefefe Tshifhiwa, Southern Africa coordinator; Dr. Gezahegne Mamo, East Africa coordinator; Dr. Fumilayo I. D. Afolayan, West Africa coordinator and Dr Yakhya Dieye, secretary. The AfVANET committee will work toward making AfVANET recognized by African governments and the African Union (AU). Furthermore, it will collaborate with experts from the World Health Organisation (WHO), the Office International des Epizooties (OIE), the African Union Centre for Disease Control (AU CDC), the African Field Epidemiology Network (AFENET), the Global Alliance for Vaccine and Immunisation (GAVI) and the Global Alliance for Livestock Vaccine and Medicine (GALVmed) to take advantage of the resources available in these regional and international organisations. To encourage the collaboration across the globe, AfVANET enjoys partnership with the IVVN. We believe that this initiative will help accelerate the role of African researchers in solving the problems of their continent and developing appropriate solutions that are in most cases different from region to region on this vast continent. Membership of AfVANET is free and we can be followed on Twitter @AAfvanet and Facebook.
